# Recent Research in Cell Stress and Microbial Infection

**DOI:** 10.3390/microorganisms10030622

**Published:** 2022-03-14

**Authors:** Quang Duy Trinh

**Affiliations:** Division of Microbiology, Department of Pathology and Microbiology, Nihon University School of Medicine, Tokyo 173-8610, Japan; trinh.duyquang@nihon-u.ac.jp

Microbial infection, including bacterial, viral, fungal, and parasitic, is a common human disease leading to various cell stresses. The interaction between host and pathogen results in cellular homeostasis alterations and triggers specific cellular stress responses. In return, microorganisms can utilize factors in cellular stress responses to facilitate their infection process. This Special Issue provides a current understanding of the latest advanced findings in cellular stress and microbial infection.

Recently published articles showed that viral infection leads to changes in cellular homeostasis of the intracellular (due to the exploitation of viral protein during the virus replication process) and/or extracellular environment (resulting from hypoxemia or other organism failure, system responses against viral infections). Various kinds of cellular stresses may be found in a specific viral infection. Zika virus could induce endoplasmic reticulum (ER) stress upon its infection in placental trophoblasts [1]. The SARS-CoV-2 infection leads to various cellular stresses, including ER stress, oxidative stress, and causes mitochondria dysfunction [2,3].

Functional subversion of the ER and a cause of its stress is found in viral infection and bacterial infection. Many bacterial effectors activating ER stress sensors have been discovered. Various bacteria evolving strategies to differentially activate ER stress sensors resulting in specific host cell responses have also been reported [4]. In bacterial infection, oxidative stress occurs during the host immune response by generating reactive oxygen species (ROS). Mitochondrial stress may occur, such as in the case of staphylococcal glycolysis [5]. Recent advanced findings suggest that bacteria such as *Staphylococcus aureus* can induce a general stress response to protect from multiple stresses, including oxidative stress, and promote tolerance of antibiotics for their survival [6].

Not only the ER stress and oxidative stress pathways are activated by bacteria and viruses, but these two have also been involved in the pathogenesis of parasites. Malaria can result in high oxidative stress in its naturally pathogenic process, either a direct result of *Plasmodium* infection of erythrocytes or a consequence of the host response to infection, along with ER stress [7]. ER stress has been found in other parasitic infections such as *Trypanosoma* or *Toxoplasma* [8]. In fungal infection, host oxidative stress, nitrosative stress, and responses of pathogenic fungi against these stresses to facilitate adaptation to the host have also been investigated [9].

There has been an increasing trend in research about the roles of cellular stress or proteins and components of the cellular stress response on microbial infection. One example of findings is that viruses, including the SARS-CoV-2, can utilize the glucose-regulated protein 78 involved in the unfolded protein responses in ER stress for their binding to target cells [10,11]. Another noted an enhancement of rubella infection in the first-trimester trophoblast cell lines under low glucose-induced ER stress conditions [12].

Consequently, discovering these relationships leads to promising drug development for therapy. The findings on the crosstalk of ER stress and the anti-viral activity came to a suggestion on using a combination of ER stress inhibitors and others to suppress the SARS-CoV-2 virus binding and replication in the target cells [3,13]. ER stress has also been suggested as a therapeutic target for relieving pathological damage of parasitosis [8]. In bacterial infection, ROS-based alternative antimicrobials targeting oxidative stress to mitigate the problem of antibiotic resistance have recently been suggested [14].

Recently advanced findings regarding the relationship between microbial infection and cellular stress will be discussed in this issue. Current investigations using advanced approaches with their results on the roles of cellular stresses in microbial infection, especially in viral infection and replication, will be presented. In addition, future research to clarify the mutual roles of cellular stress and microbial infection and promising therapy development will be discussed by invited leading authors and our research group.
